# Chronic adaptive deep brain stimulation for Parkinson’s disease: clinical outcomes and programming strategies

**DOI:** 10.1038/s41531-025-01124-7

**Published:** 2025-08-29

**Authors:** Johannes L. Busch, Jonathan Kaplan, Jennifer K. Behnke, Victoria S. Witzig, Luisa Drescher, Jeroen G. V. Habets, Andrea A. Kühn

**Affiliations:** 1 Department of Neurology, Movement Disorders and Neuromodulation Unit, Charité – Universitätsmedizin Berlin, Berlin, Germany; 2 BIH Charité Junior Clinician Scientist Program, BIH Biomedical Innovation Academy, Berlin Institute of Health at Charité – Universitätsmedizin Berlin, Berlin, Germany; 3 Neurocure Cluster of Excellence, Charité – Universitätsmedizin Berlin, Berlin, Germany; 4 German Center for Neurodegenerative Diseases (DZNE), Berlin, Germany

**Keywords:** Parkinson's disease, Parkinson's disease

## Abstract

Adaptive deep brain stimulation (DBS) dynamically adjusts stimulation amplitude based on neurophysiological feedback and may alleviate residual motor fluctuations in patients with Parkinson’s disease. However, potential clinical benefits and programming strategies remain poorly understood. We programmed eight patients with Parkinson’s disease on commercially available Dual Threshold adaptive DBS based on subthalamic beta power. Symptom severity was evaluated at home using ecological momentary assessments during two weeks of both continuous and adaptive DBS. Patients were not blinded to the stimulation mode. On the group level, overall well-being significantly improved with adaptive DBS (*p* = 0.007), and there was a non-significant trend toward enhanced general movement (*p* = 0.058). Within-subject analysis showed a significant improvement in overall well-being and general movement in three of eight patients. Six of eight patients chose to remain on adaptive DBS. Programming challenges included biomarker selection, threshold definition, and artifact-related maladaptation, for which targeted strategies are reported. Our findings support adaptive DBS as a potential option for selected Parkinson’s disease patients with persistent motor symptoms on continuous DBS. We propose a three-step programming approach to guide clinical implementation of adaptive DBS.

## Introduction

While continuous deep brain stimulation (cDBS) is an effective therapy for Parkinson’s disease (PD)^1^, residual symptoms may emerge when stimulation intensity is either too high or too low relative to the patient’s fluctuating symptom severity and medication state. This may potentially lead to overtreatment resulting in dyskinesia or undertreatment with worsened bradykinesia^2^. Consequently, adaptive DBS (aDBS) algorithms have been developed to automatically adjust stimulation amplitude based on feedback signals representative of symptom severity^3^. Specifically, since subthalamic beta activity correlates with bradykinesia and rigidity^4^, aDBS algorithms have been designed to tailor stimulation intensity to fluctuations of beta activity. As this approach has only been tested in small laboratory studies^5–7^, limited data exist to guide patient selection and programming^8–11^. With the recent commercial availability of beta-guided aDBS for PD patients, we set out to identify challenges and present solutions for aDBS programming, and report the first real-world outcomes of chronic aDBS based on subthalamic beta power.

## Results

### aDBS leads to improved overall well-being in comparison to cDBS

**Fig. 1:**
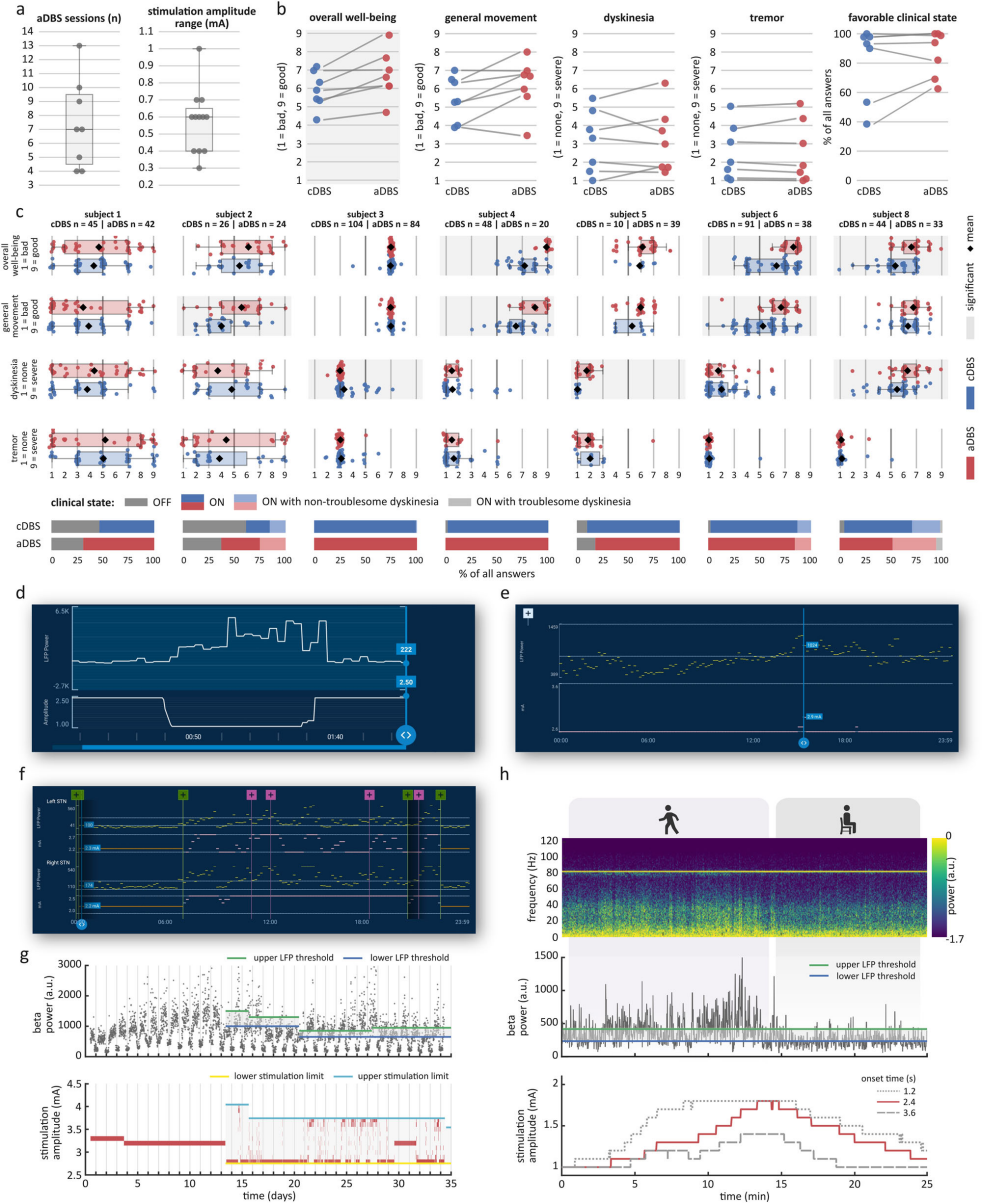
Clinical results and common programming pitfalls. **a** Distribution of the number of aDBS sessions per patient and final stimulation amplitude range per hemisphere. The gray line within each boxplot denotes the median. **b** Group-level comparisons of EMA items overall well-being, general movement, dyskinesia, and tremor. Patient-specific means are shown for cDBS and aDBS phases. Significant across-patient differences from mixed-effects models are indicated by gray shading. **c** Upper rows: Within-patient distributions of the same EMA items as in (**b**). Individual data points are shown on standard box plots, with the mean indicated by a diamond. Statistically significant within-patient differences are highlighted by gray shading. Lowermost row: Within-patient distributions of clinical status based on the PD home diary. Clinical states were binarized into favorable (ON and ON with non-troublesome dyskinesias) and non-favorable (OFF and ON with troublesome dyskinesias) for statistical analysis. Note that Subject 7 did not complete aDBS programming, so no EMA data is available for this patient. **d** Clinician Programming Tablet screenshot displaying real-time beta power (upper row) and stimulation amplitude (lower row). Beta power starts to increase following a reduction in amplitude just before the 50-second mark. **e** Chronic Timeline data from one hemisphere during active aDBS, showing 10-min averages of beta power (upper row) and stimulation amplitude (lower row). Beta power fails to consistently exceed the upper LFP threshold, resulting in amplitude remaining at the lower limit. **f** Timeline data from both hemispheres as in (**e**). In the right STN, beta power rarely drops below the lower threshold, keeping stimulation amplitude high. In contrast, the left STN shows consistent amplitude modulation throughout the day. High-resolution versions of (**d**–**f**) can be found in the Supplementary Materials. **g** Ten-minute beta power averages (upper row) and stimulation amplitude (lower row) over 35 days. The patient was on cDBS for the first 13 days and on aDBS thereafter with two days of paused aDBS after day 30. Beta power shows both intra-day variability and multi-day trends, with the latter requiring repeated LFP threshold adjustments. **h** Upper row: Spectrogram of LFP data during aDBS while the patient was walking (first 15 min) and resting (last 10 min), showing elevated broadband power during movement. Middle row: Real-time beta power from the Percept system showing increased activity during walking. Lower row: Stimulation amplitude (red line) increases during walking in response to real-time beta power crossing the upper LFP threshold and decreases after the patient stops moving. Simulated amplitude traces (dashed gray lines) show variation in the stimulation amplitude response based on different onset times.

Patients attended 7.8 ± 3.7 (mean ± standard deviation; range 4–13) programming visits until a satisfactory aDBS configuration was achieved or until they reverted to cDBS (Fig. 1a, left). In patients who remained on aDBS and were stimulated on the same contact as during cDBS, the upper stimulation limit was on average 0.23 mA higher (mean upper limit: 2.28 mA) and the lower limit 0.33 mA lower (mean lower limit: 1.71 mA) than the amplitude used during cDBS (mean 2.04 mA). The final average range of all patients who stayed on aDBS was 0.58 ± 0.19 mA (range 0.3–1 mA) (Fig. 1a, right). Pulse width and stimulation frequency remained stable between conditions, except in one patient whose frequency had previously been reduced to 85 Hz to prevent dyskinesia. With aDBS, we were able to increase the frequency to the standard 125 Hz, achieving better tremor control while also reducing dyskinesia intensity.

Clinical outcomes were acquired at home using ecological momentary assessments (EMA). The average number of EMA collection days was 13.6 ± 5.1 during cDBS and 17.3 ± 4.9 during aDBS. The average EMA completion rate per patient was 59.2% for cDBS and 49.6% for aDBS, with a total of 52.6 questionnaires completed in the cDBS condition and 40 in the aDBS condition per patient on average.

On the group level, overall wellbeing improved from 5.92 ± 1.01 to 6.73 ± 1.33 points (mean ± standard deviation of subject-specific means; *β* = 0.8, *t* = 3.193, *p* = 0.007) and there was a trend for enhanced general movement (5.47 ± 1.22 to 6.2 ± 1.44, *β* = 0.7, *t* = 2.271, *p* = 0.058) (Fig. 1b). There was no difference in dyskinesia (cDBS: 3.12 ± 1.7, aDBS: 3.18 ± 1.76, *β* = 0.03, *t* = 0.108, *p* = 0.988) and tremor (cDBS: 2.54 ± 1.51, aDBS: 2.56 ± 1.67, *β* = 0.002, *t* = 0.002, *p* = 0.988). A favorable clinical status (ON or ON with non-troublesome dyskinesia) was reported slightly more often during aDBS (cDBS: 81.5 ± 25%, aDBS: 87 ± 16%; *β* = 0.37, *t* = 1.19, *p* = 0.23).

On an individual basis, overall well-being improved in all patients during aDBS, reaching statistical significance in three (Fig. 1c). General movement improved in five of seven patients, with significant effects in three. Dyskinesia severity decreased in four patients (one significant) and increased in three (two significant). Tremor symptoms lessened in five patients and worsened in two, though none of these changes were statistically significant. All patients except subject 5 (more OFF phases during aDBS) shifted towards more favorable clinical states during aDBS compared to cDBS, though this was not statistically significant within individuals. Distributions of the remaining items of the EMA questionnaire are reported in Supplementary Fig. 1.

One patient (Subject 7) chose to return to cDBS before programming could be completed. They had only minor residual complaints during cDBS, primarily related to fine motor control, and found additional programming visits too burdensome relative to the expected benefit. Two weeks after the final follow-up (post-EMA collection), six of the remaining seven patients chose to stay on aDBS. Subject 6 reverted to cDBS due to persistent gait impairment with recurring festination episodes, which did not improve with aDBS. Still, EMA data indicated improved overall well-being and enhanced general movement during aDBS in this subject.

### Challenges and solutions to set up aDBS in PD patients

Despite varying clinical challenges across patients, we identified common issues and developed strategies to address them.

### Contact and beta peak selection

**Fig. 2:**
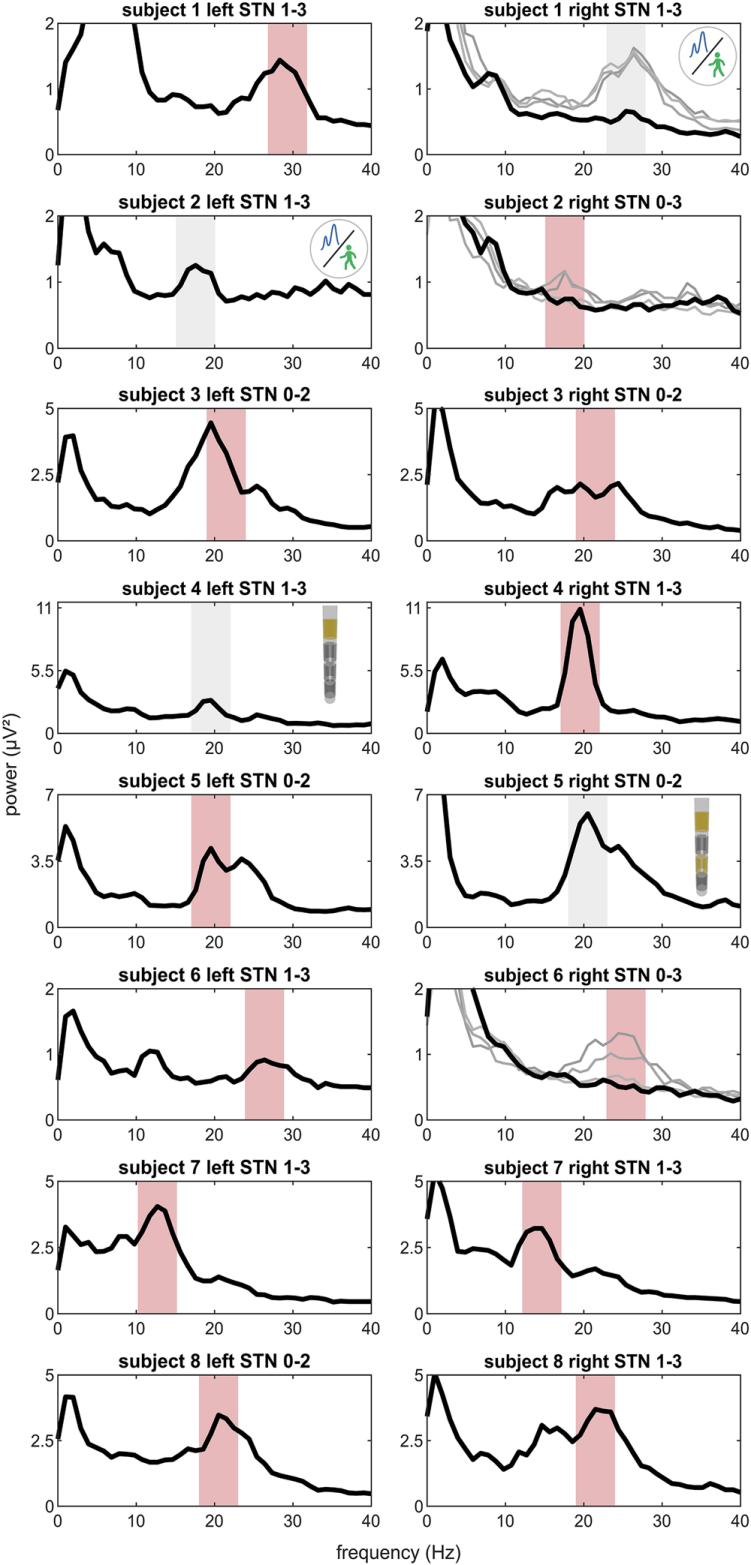
Power spectra and programmed sensing ranges. Power spectra obtained during the Timeline setup using the Signal Test modality of the Percept are shown for both hemispheres of each subject. The selected sensing range is highlighted in red. Gray bands indicate the presence of a beta peak, however, sensing was not activated in the respective STN for the following reasons: poor signal-to-noise ratio in subjects 1 and 2 and sensing-incompatible stimulation contacts in subjects 4 and 5. The recording channel used is indicated in each subplot title. In the right STN of subjects 1, 2, and 6 no clear beta peak was identified during setup while patients were ON medication. For these STNs, gray traces show archival Signal Test data acquired after overnight withdrawal of dopaminergic medication (all channels).

Before aDBS activation, sensing contacts and respective beta peaks must be selected. In 3/16 hemispheres, no beta peak was visible (Fig. 2, Supplementary Fig. 4). Our patients attended the preparation visit taking regular medication, which may have obscured peak detection in these cases. To ensure the Timeline tracks an existing peak, we reviewed prior OFF medication Signal Test local field potential (LFP) data and recommend repeating it in the OFF medication state (Fig. 2). Nevertheless, unilateral sensing was enabled in 2/8 patients due to a suboptimal signal-to-noise ratio in the other STN (Fig. 2). Double beta peaks were observed in 4/16 subthalamic nuclei (STN). Given that beta-based aDBS relies on control signal modulation, continuous test stimulations and assessing medication-induced beta power modulation helped to identify the most responsive peak in these cases (Fig. 1d). In 2/8 patients, sensing-compatible contacts demonstrated suboptimal clinical efficacy (Fig. 2). To allow for flexible contact selection, we enabled unilateral sensing in these patients. In total, unilateral sensing was activated in 4/8 patients.

### Selection of LFP thresholds and stimulation limits

Continuous Timeline data acquisition over several days enabled us to review beta band modulation per patient before setting LFP thresholds to the 25th and 75th percentiles of daytime beta power per manufacturer guidance. As beta power modulation varied considerably between patients, final LFP thresholds showed strong inter-individual variance (upper threshold: 1011 ± 924, range: 225–3160; lower threshold: 691 ± 843, range 100–2970; spread between upper and lower threshold: 320 ± 219, range: 25–850; all patients who concluded aDBS programming). In Subject 7, no Timeline data was available and LFP thresholds were set using in-clinic “BrainSense Streaming” (Streaming) data. However, these thresholds proved too high at follow-up with stimulation amplitude remaining at the lower limit. As the patient was still experiencing OFF motor symptoms, in-clinic Streaming data likely misrepresented long-term beta power levels (Fig. 1e). In Subject 2 large LFP outliers complicated Timeline interpretation because high power values distorted the scale, compressing the true feedback signal (Supplementary Fig. 4). This was prevented by setting arbitrary thresholds for the preparation phase during cDBS. Determining stimulation limits in the ON medication state underestimated the lower limit in 4/8 patients in the aDBS optimization phase when thresholds were not adequately tracking beta power yet. To prevent OFF symptoms, we recommend evaluating the minimum effective stimulation in the OFF medication state for such cases.

### Parameter adaptation during optimization phase

Optimization visits ensured that LFP thresholds allowed stimulation amplitude to track beta power changes, and that amplitude limits provided adequate symptom control. Adverse events after initial aDBS setup necessitating parameter adjustments included under-stimulation with increased motor symptoms during daytime, prolonged morning OFF periods, nocturnal OFF dystonia, over-stimulation with worsened dyskinesia, worsened dysarthria and more rapid motor fluctuations.

In 6/16 hemispheres, stimulation remained stuck at either lower or upper stimulation limit on the first optimization visit, which required adjustment of LFP thresholds. For example, in Subject 6, stimulation adapted well in the left STN but remained at the upper limit most of the day in the right hemisphere (Fig. 1f). To address this, we increased right STN LFP thresholds from 110–270 to 250–500. In 3/16 hemispheres, multi-day beta power trends complicated LFP threshold adjustments, leading to intermittent inadequate stimulation adaptation (Fig. 1g).

If stimulation adapted well but hyperkinetic or hypokinetic episodes persisted, amplitude limits required refinement. In Subject 6, despite proper adaptation between 2.2 and 2.7 mA in the left hemisphere, hypokinetic episodes continued. Timeline events showed tremor reoccurrence at the lower limit, especially before the next medication dose. To improve symptom control, we raised the lower limit to 2.4 mA.

Dyskinetic periods occurred during optimization in 4/8 patients despite proper stimulation adaptation. In such cases, dyskinesia-induced movement artifacts must be considered as a potential source of false-positive high beta power, which may trigger an increase in stimulation amplitude and further exacerbate dyskinesia. Patient-specific events helped to identify such periods in Timeline data. In Subject 2, an in-clinic Streaming assessment confirmed that walking-related power increases were primarily driven by artifacts (Fig. 1h). To mitigate this, we took three steps: (1) we enabled unilateral sensing in the less artifact-prone right hemisphere, (2) lowered the upper stimulation limit to reduce stimulation-induced dyskinesia, and (3) increased onset duration to 3.6 s to reduce the sensitivity to brief artifact-driven transients.

## Discussion

We report on eight chronically stimulated PD patients who were programmed with beta power-based Dual Threshold aDBS using a recently introduced commercial DBS system. Seven of these patients completed aDBS programming, and six of these patients have remained on aDBS for over four months. In a real-world setting, aDBS significantly improved overall well-being compared to optimized cDBS in three of seven patients. This was reflected in patient-specific changes across motor domains, with general movement improving significantly in three of seven patients. Importantly, improved general movement was not linked to worsened dyskinesia, suggesting that aDBS enhanced motor function without increasing dyskinesia risk. This aligned with a trend towards more favorable clinical states, although dyskinesia increased during aDBS in two out of seven patients. Interestingly, despite being present in five patients, tremor did not worsen with aDBS although beta band activity is considered a more specific biomarker for bradykinesia and rigidity^12^.

**Fig. 3:**
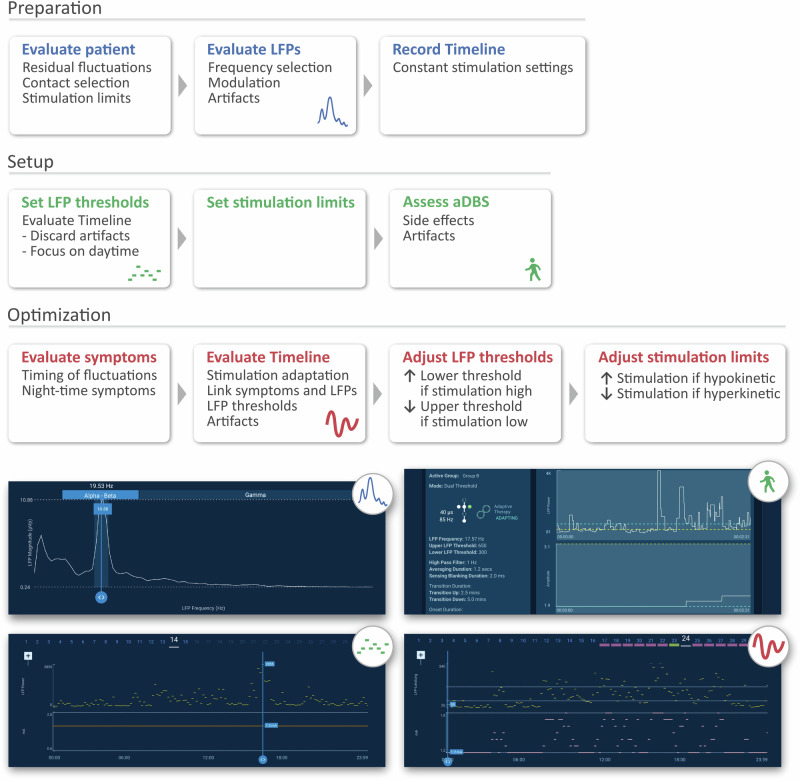
Proposal for an aDBS programming pipeline. During a preparatory visit, the patient’s clinical characteristics are evaluated, and chronic biomarker recording is configured. After sufficient at-home recording time, the aDBS algorithm is set up based on the observed biomarker dynamics and tested for acute side effects, e.g., due to artifacts triggering stimulation. Repeated optimization visits ensure that stimulation adapts over time in response to clinical fluctuations and that the selected stimulation limits are sufficient to provide symptom control in both the hypo- and hyperdopaminergic state without inducing side effects.

We propose a three-step clinical workflow that aligns with previously proposed strategies for closed-loop neuromodulation^3^ (Fig. 3). While our approach benefits from an already optimized cDBS setup, it might also be applied to DBS-naïve patients. First, during a preparation visit contacts and beta peaks should be selected to enable chronic biomarker sensing during a phase with cDBS. Contact selection is based on clinical assessment, beta peak presence, and artifact susceptibility, ideally converging on the same contact^13^. Beta peak identification can be improved by recording OFF medication LFPs^8^ and assessing stimulation- or medication-induced modulation^14^ (Fig. 1d, Supplementary Fig. 4). Of note, a low beta peak that extends into the alpha range might still be suitable to inform aDBS^8,15,16^. If clinical constraints require stimulation on a non sensing-compatible contact or if LFPs lack a clear beta peak or are artifact-prone, unilateral sensing may be used to control stimulation on both hemispheres. To improve Timeline interpretation, patients should avoid manual amplitude adjustments or program switches. Additionally, setting LFP thresholds during Timeline setup helps prevent scale distortions from outliers. Patient-triggered Events can aid interpretation of symptoms in respect to Timeline data.

Second, the aDBS algorithm is set up. LFP thresholds should be based on Timeline data, which reflects long-term beta power^17^, as short-term Streaming data proved to be unreliable. Initial threshold values based on percentiles are a first approximation, refined later based on clinical response. Stimulation limits must be chosen carefully to prevent over- or under-stimulation. We recommend evaluating the upper stimulation limit in the relative OFF medication state and selecting an amplitude that suppresses OFF motor symptoms. Conversely, we advise assessing the lower stimulation limit in the ON state. The corresponding amplitude should not induce dyskinesia. However, in some cases adjustments to stimulation limits may be necessary to safeguard against situations in which there is a mismatch between the clinical state and stimulation amplitude. For example, movement artifacts due to ON-state dyskinesia can lead to an increase in beta power, which in turn triggers stimulation to ramp up. For such cases, the upper stimulation limit should also be assessed while ON medication. This ensures that the corresponding stimulation amplitude does not further aggravate dyskinesia. Once adaptation has been refined and no mismatch can be detected, the stimulation limits may be gradually reset to their original values.

Finally, the aDBS program is iteratively optimized to ensure LFP thresholds capture beta power dynamics, allowing stimulation amplitude to adapt. If stimulation remains at the lower limit, the upper LFP threshold should be lowered, particularly to enable ramp-up after sleep-related beta suppression^17^. Conversely, if stimulation stays at the upper limit, the lower LFP threshold should be raised. The optimal ratio between low and high stimulation amplitudes is patient-specific. Ideally, optimal thresholds lead to stimulation amplitude fluctuations which follow medication cycles, increasing in the OFF state and decreasing in the ON state. If adaptation occurs but hypo- or hyperkinetic symptoms persist, it must be determined whether they align with periods of low or high stimulation. Stimulation limits should then be adjusted accordingly.

Despite aDBS not worsening overall well-being in any patient who completed programming, one eventually reverted to cDBS due to persistent gait impairment. Overall, gait impairment and mild hypokinetic symptoms were more challenging to address with Dual Threshold aDBS in our cohort than residual and significant motor fluctuations. However, given the small sample size, we cannot make a clear suggestion in regards to the patient population that might benefit most from aDBS. As an advanced therapy option, aDBS might be employed particularly in centers with sufficient expertise and resources for the time-intensive programming it requires.

Our findings have implications for the further development of aDBS algorithms. For example, to improve robustness of the closed-loop system against movement artifacts, an outlier rejection mechanism may be employed. In the future, a skull-mounted pulse generator may significantly reduce artifact load^18^. Further research needs to characterize the differential contributions of non-oscillatory and oscillatory components to long-term biomarker fluctuations, which may improve chronic tracking of clinical states^19^. Finally, incorporating spectral markers outside the beta range will likely improve motor state decoding as for example finely tuned or entrained gamma band activity for dyskinesia monitoring^20,21^.

This report is limited by the restricted sample size and the non-randomized and open-label design. As such, rigorously designed clinical studies need to determine whether a clinical benefit can be observed in a larger population and to identify sub-groups of patients who are most likely to benefit from aDBS. Future studies should also quantitatively assess changes to stimulation-related side effects such as dysarthria, which was beyond the scope of this study. Moreover, this case series relied on patient-reported outcomes, lacking objective symptom assessments. Furthermore, since we used a commercially available aDBS system, our choice of biomarkers and control policies was limited. As a result, we were unable to incorporate recent advances in closed-loop DBS, such as the use of gamma frequency biomarkers^22,23^.

In summary, our study suggests that Dual Threshold aDBS constitutes a potential therapy option in PD patients with clinically optimized cDBS, who continue to experience residual motor symptoms. Albeit being resource-intensive, our proposed guidelines to streamline aDBS programming may facilitate adoption of aDBS in clinical practice.

## Methods

### Participants

This study was conducted in accordance with the Declaration of Helsinki and was approved by the institutional review board at Charité – Universitätsmedizin Berlin. All patients provided written informed consent. Eight PD patients implanted with a sensing-enabled DBS system (Percept, Medtronic, Minneapolis, MN, USA) targeting the subthalamic nucleus were included (three female; mean ± SD age: 60.75 ± 11.51 years; disease duration: 14.12 ± 3.36 years; time since implantation 2.5 ± 1.07 years). All patients underwent multiple clinical programming visits and were on stable cDBS settings. Patient selection was not guided by specific clinical or electrophysiological characteristics to allow for outcome variability. Clinical details are shown in Table 1.

**Table 1:** Demographic and clinical details

								STN left			STN right					
#	Age (years)	Sex	Disease duration (years)	Time since surgery (years)	MDS UPDRS III Med OFF Pre-surgery	MDS UPDRS III Med OFF Stim ON 12-months Post-surgery	Chief complaint	Stimulation amplitude limits (mA)	LFP thresholds (µVp)	Beta sensing peak (Hz)	Stimulation amplitude limits (mA)	LFP thresholds (µVp)	Beta sensing peak (Hz)	Concluded aDBS setup	Stayed on aDBS	aDBS sessions (n)
1	74	F	14	4	42	28	Dyskinesia	1.2–1.8	105–270	29.30	1.2–1.8	Unilateral	Unilateral	Yes	Yes	5
2	68	F	10	4	68	20	Dyskinesia	1.8–2.2	Unilateral	Unilateral	1.1–1.7	200–225	17.58	Yes	Yes	13
3	66	F	10	2	50	16	Tremor	1.8–2.8	1725–2575	21.48	2.2–2.8	500–1000	21.48	Yes	Yes	7
4	42	M	17	2	90	42	Tremor	2.1– 2.5	Unilateral	Unilateral	2.1–2.5	320–720	19.53	Yes	Yes	4
5	44	M	12	2	30	32	Peak-dose dystonia	1.4–2	250–700	19.53	2.1–2.4	Unilateral	Unilateral	Yes	Yes	10
6	62	M	17	3	55	20^a^	Gait impairment	2.4–2.7	100–280	26.37	2 - 2.5	220–400	25.39	Yes	No	7
7	64	M	19	2	71	61	Fine motor control	2.8–3.6	2970–3160	12.70	2.8 - 3.6	700–1100	14.65	No	No	4
8	66	M	14	1	72	16^a^	Dyskinesia	2.8–3.5	550–750	20.51	2.8 - 3.5	650–950	21.48	Yes	Yes	9

*MDS UPDRS III* Movement Disorders Society sponsored version of the Unified Parkinson’s disease Rating Scale Part III, *LFP* Local field potentials, *STN* Subthalamic nucleus, *aDBS* adaptive deep brain stimulation.

^a^Medication ON.

### aDBS setup procedure

Patients attended a preparation visit, an aDBS setup session, and multiple follow-up visits. During the preparation visit, cDBS settings were reviewed and adjusted if sensing-incompatible contacts or stimulation modes were active (7/16 STNs)^24^. This was mainly due to complex parameter configurations that had been introduced during earlier adjustments of cDBS due to remaining motor symptoms or side effects such as dyskinesia. A short-term clinical evaluation ensured efficacy and absence of severe side effects. Subthalamic LFPs were recorded at rest (ON medication, OFF stimulation; “Signal Test”). Power spectra were screened for an oscillatory peak within the beta frequency range (13–30 Hz) using “BrainSense Setup”. If no peak was visible, prior OFF medication Signal Tests were analyzed. Of note, electrophysiology-guided contact selection using the “Electrode Identifier” was not employed. A five hertz band around each beta peak was selected. “BrainSense Timeline” (Timeline) was activated to chronically record 10-minute band-power averages^24^. Patients could record symptom-specific events to relate Timeline data to clinically relevant incidents (e.g., periods of dyskinesia). After that, patients were discharged with the option to revert to their previous DBS program if clinical deterioration occurred. Two weeks later, patients returned for aDBS setup (ON medication). Manufacturer engineers assisted with initial sessions. Timeline data was clinically related to patient reports of medication intake to ensure that beta power tracks medication cycles. All patients were configured to Dual Threshold mode, which adjusts stimulation amplitude between two limits on a scale of minutes^6^. Lower and upper LFP thresholds were set to the 25th and 75th percentile of daytime beta power based on Timeline data. Stimulation limits were based on clinically determined lowest effective and maximum tolerable amplitudes. Other aDBS parameters followed manufacturer defaults^8^. Each patient had at least two follow-ups, during which the clinical course was assessed, and Timeline data were reviewed for stimulation adaptation. We defined a satisfactory aDBS configuration as one in which stimulation properly adapts to beta activity without remaining fixed at either amplitude limit, and where patients show no severe symptoms related to over- or understimulation. Patients were not blinded to the stimulation mode.

### Clinical outcome assessment

Outcome measures were collected as ecological momentary assessments (EMA)^25^ at home on a smartphone app (Virgobit, Münster, Germany). Participants completed an identical symptom questionnaire six times daily at semi-random intervals with a 15-min response window to prevent completion- and recall-bias. EMA data were collected during two phases: (1) cDBS, from the preparation visit until aDBS setup, and (2) with aDBS, beginning at the final follow-up and lasting about two weeks. The analysis focused on selected items from the full questionnaire: overall well-being (item 1), general movement (item 6), tremor (item 7), dyskinesia (item 8, all with scale: 1–9), and a clinical status classification (item 11, see Supplementary Materials for details). Group-level effects were assessed using linear mixed-effects models for continuous variables, and a generalized linear mixed-effects model with a logit link function for the clinical status, each with random intercepts and slopes for participants. For individual-level analyses, the Mann–Whitney U test was applied to continuous outcomes, and Fisher’s exact test was used for the binary clinical status item. False Discovery Rate corrected *p*-values are reported in both cases to account for multiple comparisons.

## Supplementary information

Supplementary Material

## Data Availability

The data that support the findings of this study are available from the corresponding author on reasonable request.
