# Supplementary material for: Chronic adaptive deep brain stimulation for Parkinson’s disease: clinical outcomes and programming strategies
Source: NPJ Parkinsons Dis. 2025 Aug 29;11:264. doi: 10.1038/s41531-025-01124-7 (PMC12397205; doi:10.1038/s41531-025-01124-7)
Supplement: Supplementary file 1 — Supplementary Material [file 41531_2025_1124_MOESM1_ESM.docx]

Supplementary material to:

Chronic adaptive deep brain stimulation for Parkinson’s disease: Clinical outcomes and programming strategies

## Ecological momentary assessments and motor state classification

The full ecological momentary assessment (EMA) questionnaire comprises 15 items and was developed through a patient engagement process ^1^. The translated items are listed in Supplementary Table 1; the original version was administered in German. Unless otherwise specified, items were rated on a scale from 1 ("totally disagree") to 9 ("totally agree"). Item 11 represents a clinical status classification based on the PD home diary ^2^. We report the proportion of responses indicating either favorable (ON, ON with non-troublesome dyskinesia) or unfavorable (OFF, ON with troublesome dyskinesia) states. Two check-in calls were performed to promote adherence and to collect informal feedback.

| **Item** | **English Translation** |
| --- | --- |
| 1 | I feel well. |
| 2 | I can handle my tasks with motivation. |
| 3 | I feel sad and down. |
| 4 | I feel energized vs. tired. |
| 5 | I am experiencing at least one of the following symptoms: I am risk-taking, I act impulsively, I feel restless. |
| 6 | At the moment, I can move well. |
| 7 | I am experiencing tremor. |
| 8 | I'm experiencing excessive or uncontrolled movements (dyskinesia). |
| 9 | I can walk well. |
| 10 | I can use my hands well. |
| 11 | My current therapy state is: (Multiple choice: OFF, ON, ON with non-troublesome dyskinesia, ON with troublesome dyskinesia) |
| 12 | I have taken Parkinson’s medication in the past 30 minutes. (Multiple choice: yes, no) |
| 13 | Where are you? (Multiple choice: work, at home, indoor public place, outdoor public place, in transit) |
| 14 | Who are you with? (Multiple choice: alone, in company) |
| 15 | This questionnaire interfered with my activities. |

**Supplementary Table 1.** EMA questionnaire.

##
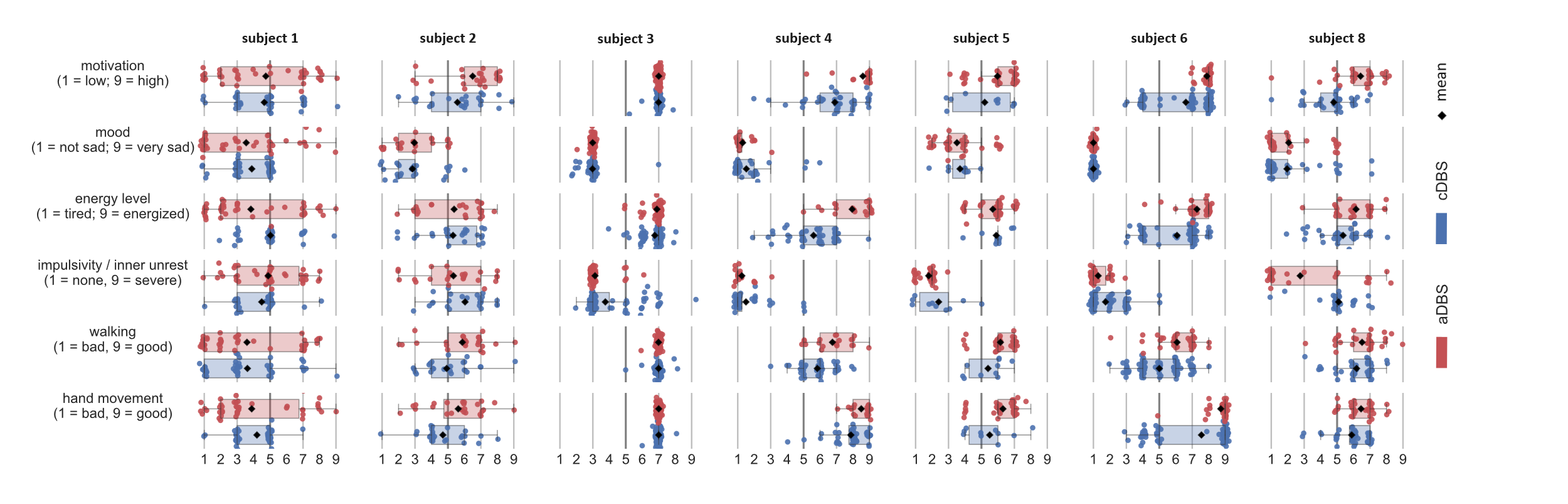
Supplementary Figures

**Supplementary Figure 1.** Additional EMA questionnaire items per subject. On the group level, motivation improved from 5.92 ± 1 to 6.73 ± 1.27 points (mean ± standard deviation of subject-specific means). In contrast, average mood changed only slightly, from 2.56± 1.1 to 2.48 ± 1.04 (where lower scores reflect better mood) points. Energy levels increased during aDBS (6.17± 1.37) compared to cDBS (5.72± 0.58). Impulsivity and restlessness decreased with aDBS (2.92± 1.66) versus cDBS (3.57 ± 1.75). Walking performance improved from 5.42± 1.07 to 5.99 ± 1.13 points, and hand movement performance was slightly better during aDBS (6.64 ± 1.67) compared to cDBS (6.1 ± 1.42). No statistical tests were conducted for this descriptive comparison.


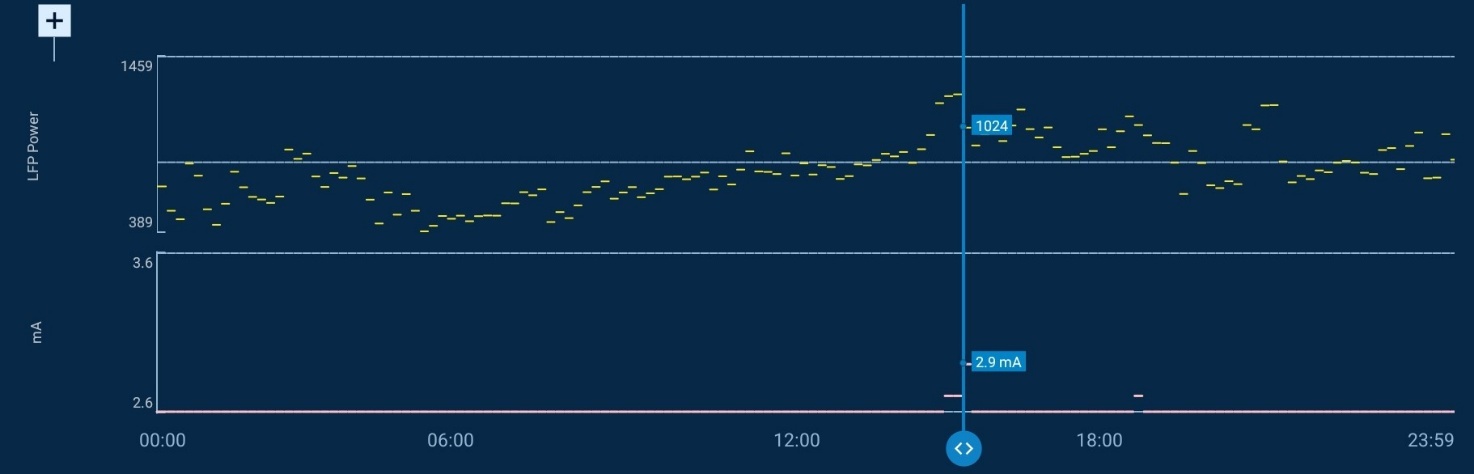


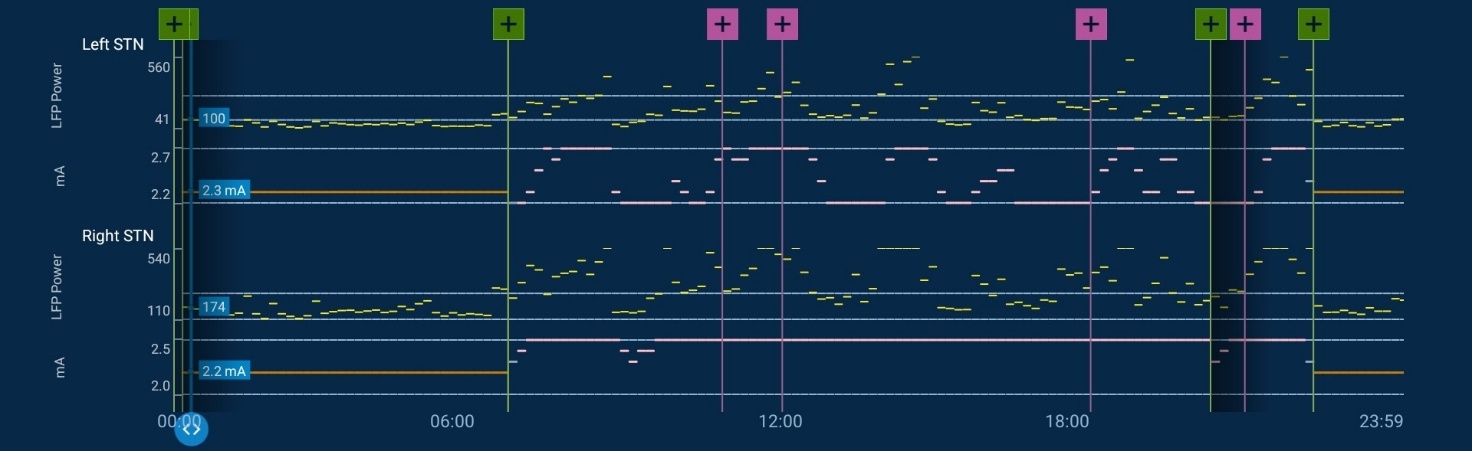

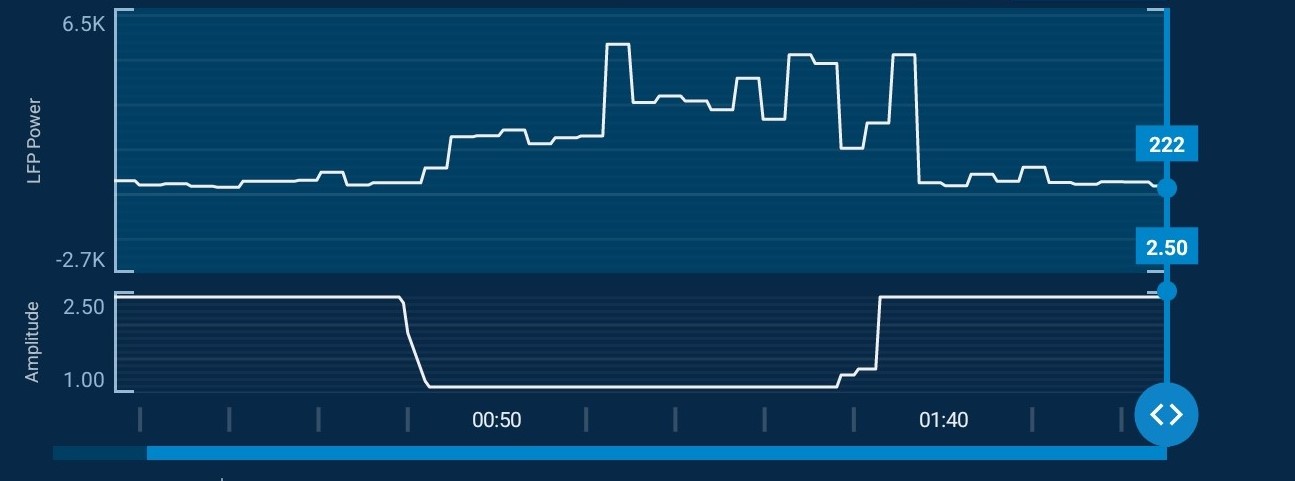
**Supplementary Figure 2.** Tablet screenshots as in Fig. 1d, 1e and 1f in a larger format.


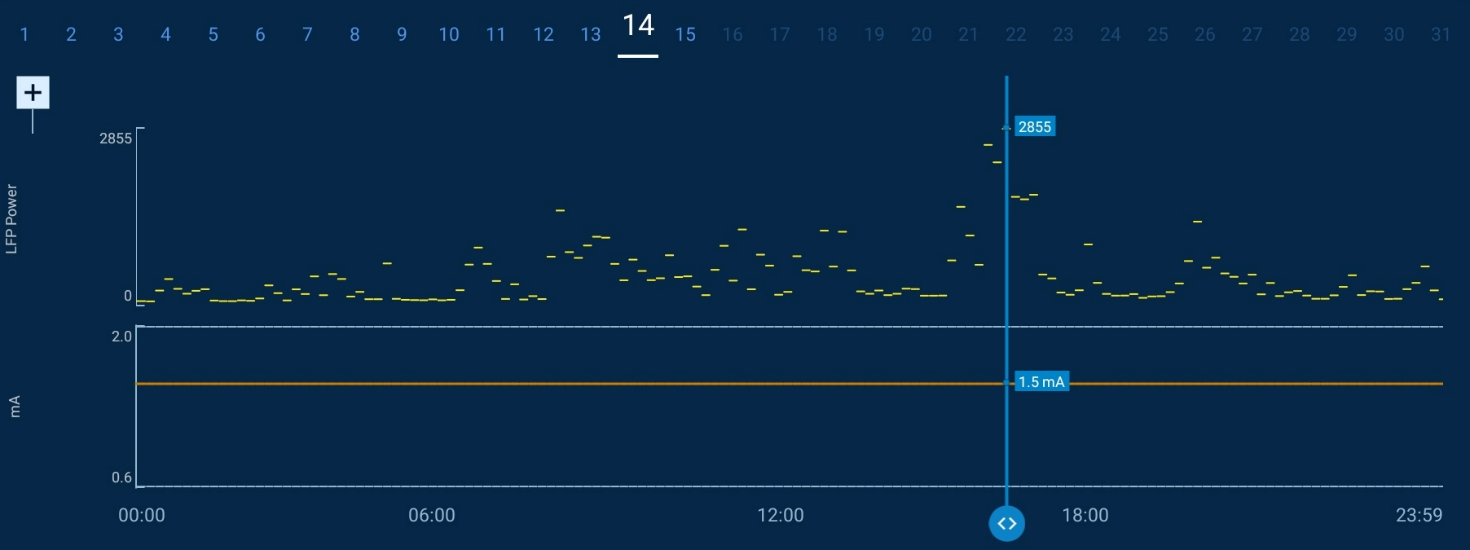

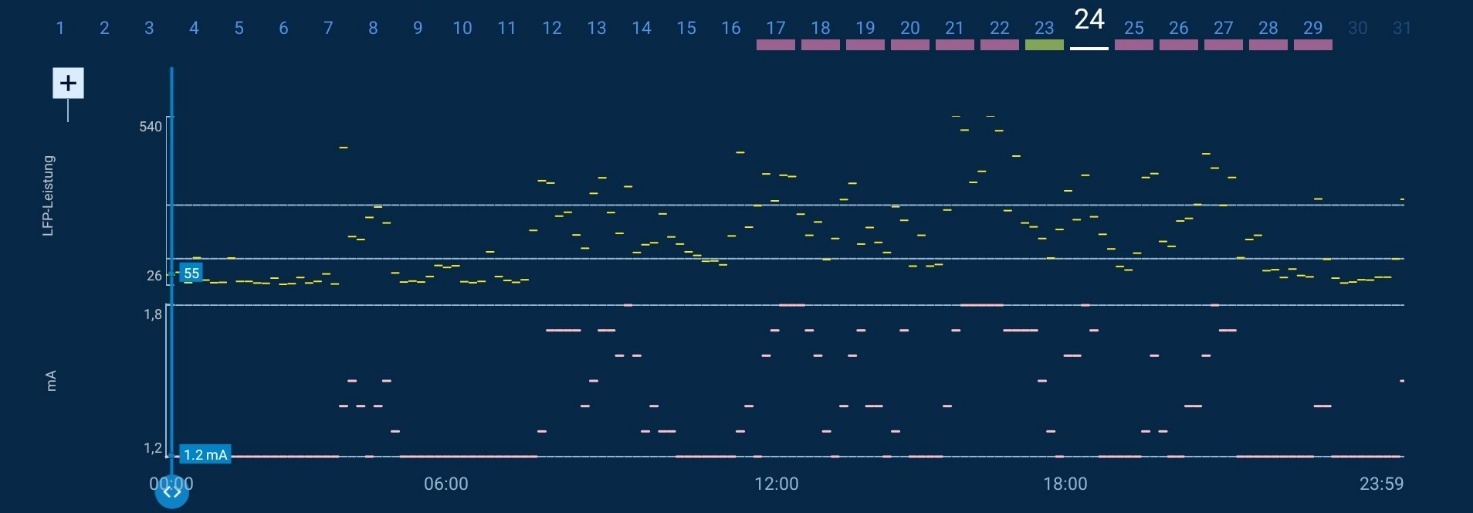

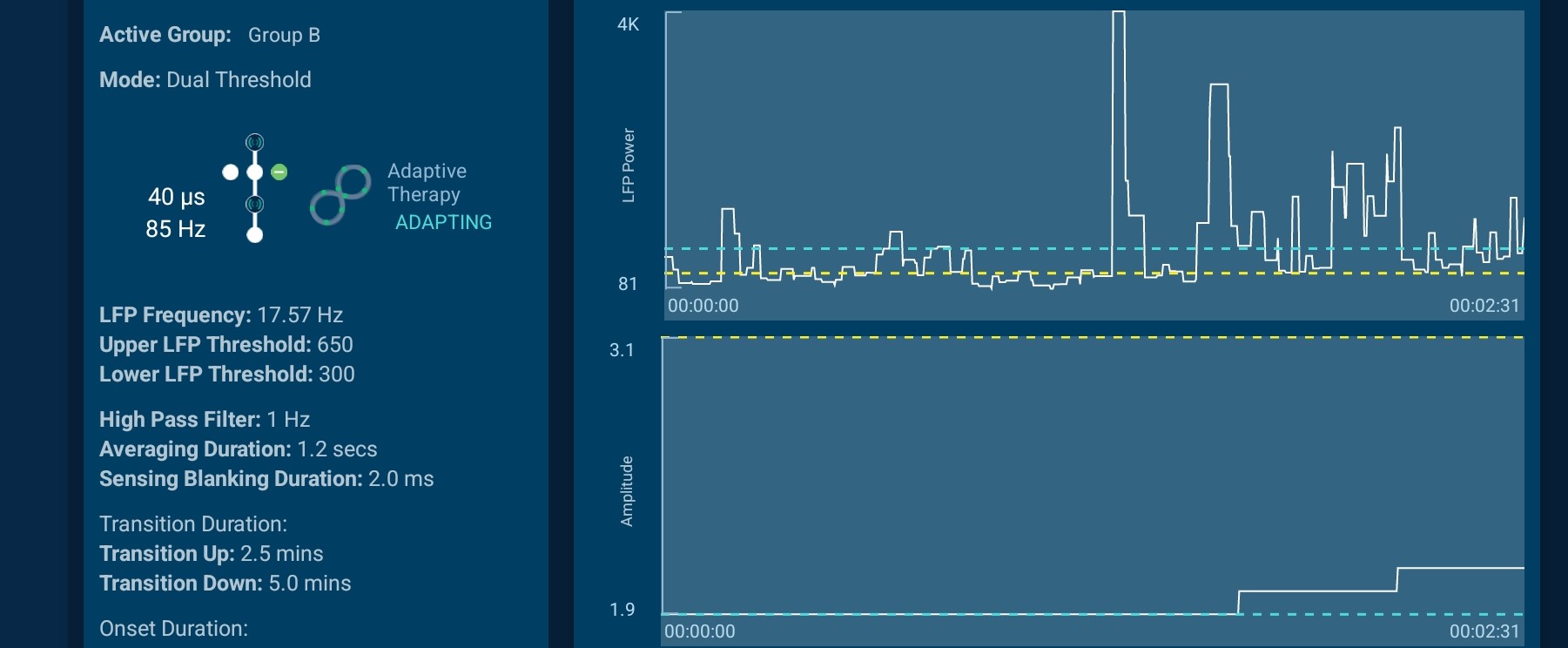

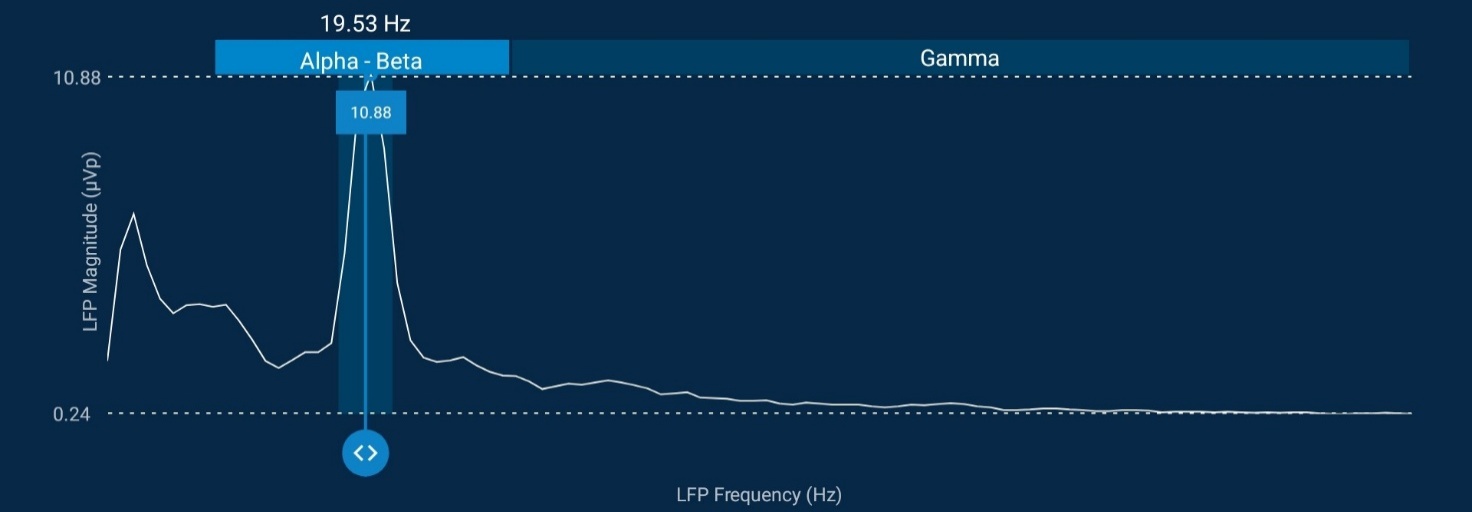
**Supplementary Figure 3.** Tablet screenshots as in Fig. 3 in a larger format.


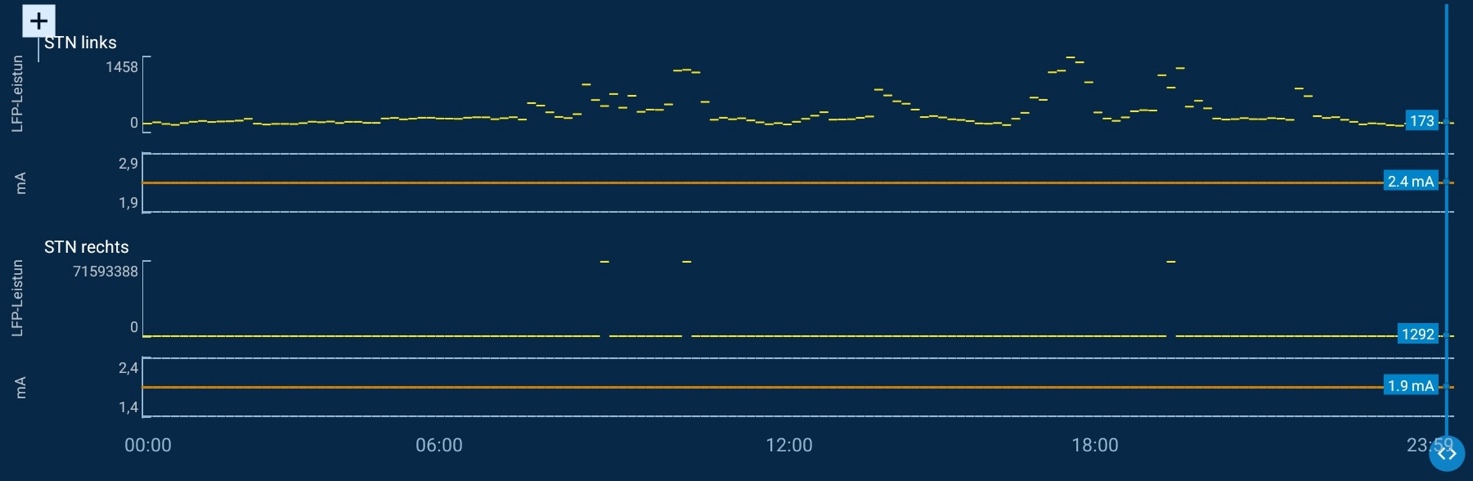

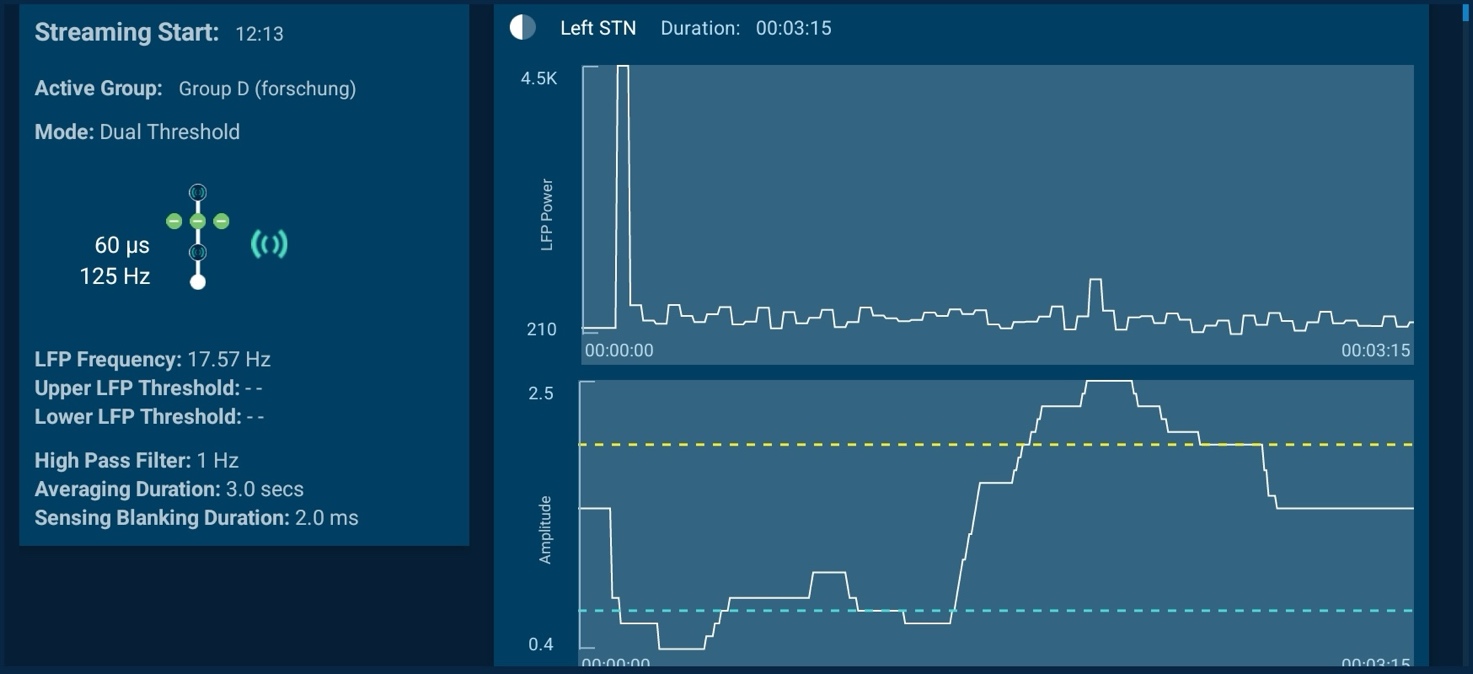


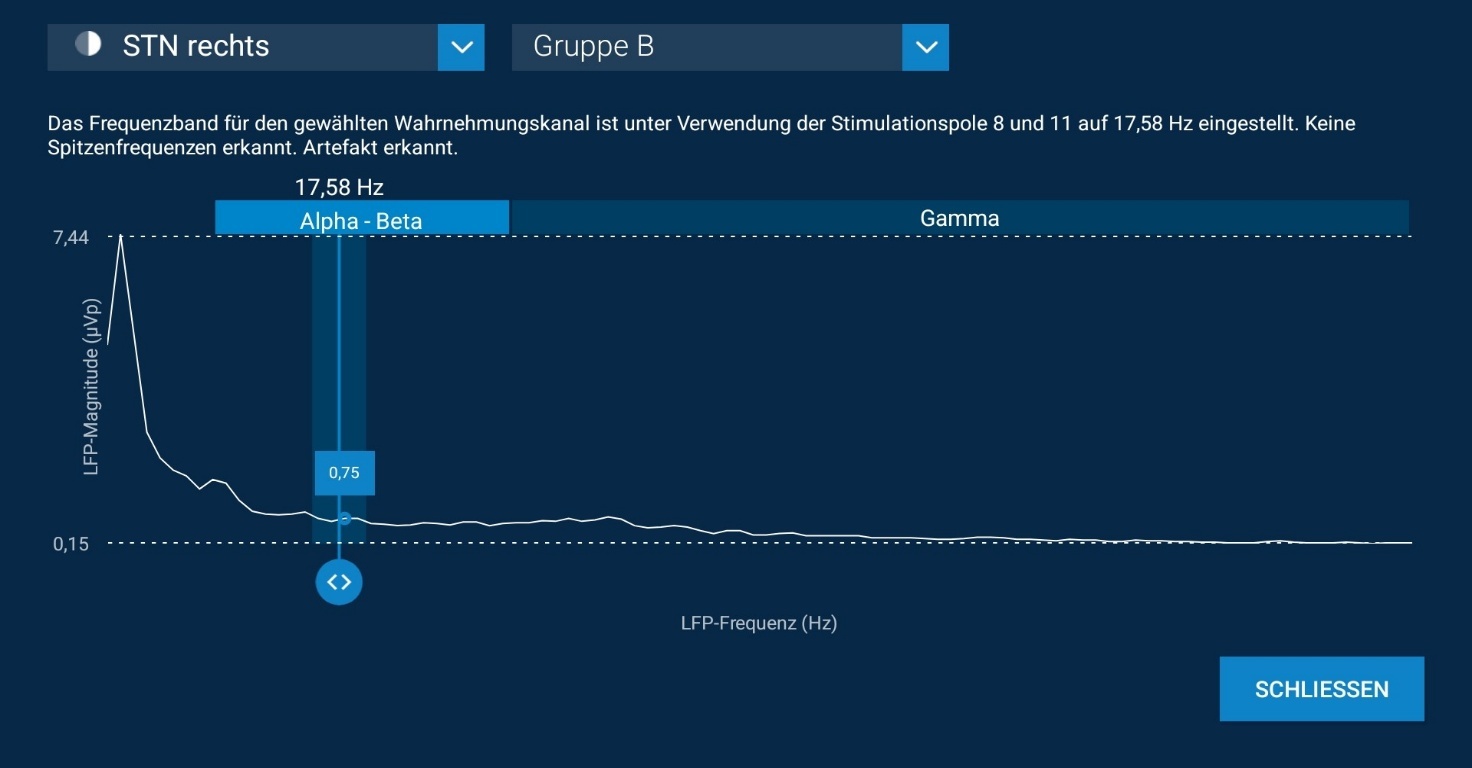
**Supplementary Figure 4.** Tablet screenshots of a Signal Test spectrum without a clear beta peak (top) and a BrainSense Streaming showing absent power suppression with increasing stimulation amplitude (middle). At the bottom, extreme outliers in the right STN – likely due to brief device-related artifacts rather than movement related power elevations – inflate the Timeline scale and obscure true signal variation.

## References

1. Feldmann, L. K., Roudini, J., Kühn, A. A. & Habets, J. G. V. Improving naturalistic neuroscience with patient engagement strategies. *Front. Hum. Neurosci.* **17**, 1325154 (2024).

2. Hauser, R. A. *et al.* A Home Diary to Assess Functional Status in Patients with Parkinson’s Disease with Motor Fluctuations and Dyskinesia: *Clinical Neuropharmacology* **23**, 75–81 (2000).
